# Design Principles of Pancreatic Islets: Glucose-Dependent Coordination of Hormone Pulses

**DOI:** 10.1371/journal.pone.0152446

**Published:** 2016-04-01

**Authors:** Danh-Tai Hoang, Manami Hara, Junghyo Jo

**Affiliations:** 1 Asia Pacific Center for Theoretical Physics, Pohang, Gyeongbuk 36763, Korea; 2 Department of Natural Sciences, Quang Binh University, Dong Hoi, Quang Binh 510000, Vietnam; 3 Department of Medicine, The University of Chicago, Chicago, IL 60637, United States of America; 4 Department of Physics, Pohang University of Science and Technology, Pohang, Gyeongbuk 36763, Korea; La Jolla Institute for Allergy and Immunology, UNITED STATES

## Abstract

Pancreatic islets are functional units involved in glucose homeostasis. The multicellular system comprises three main cell types; *β* and *α* cells reciprocally decrease and increase blood glucose by producing insulin and glucagon pulses, while the role of *δ* cells is less clear. Although their spatial organization and the paracrine/autocrine interactions between them have been extensively studied, the functional implications of the design principles are still lacking. In this study, we formulated a mathematical model that integrates the pulsatility of hormone secretion and the interactions and organization of islet cells and examined the effects of different cellular compositions and organizations in mouse and human islets. A common feature of both species was that islet cells produced synchronous hormone pulses under low- and high-glucose conditions, while they produced asynchronous hormone pulses under normal glucose conditions. However, the synchronous coordination of insulin and glucagon pulses at low glucose was more pronounced in human islets that had more *α* cells. When *β* cells were selectively removed to mimic diabetic conditions, the anti-synchronicity of insulin and glucagon pulses was deteriorated at high glucose, but it could be partially recovered when the re-aggregation of remaining cells was considered. Finally, the third cell type, *δ* cells, which introduced additional complexity in the multicellular system, prevented the excessive synchronization of hormone pulses. Our computational study suggests that controllable synchronization is a design principle of pancreatic islets.

## Introduction

Living systems have structural designs for their functional demands, which has been referred to as *symmorphosis* [1]. The islets of Langerhans in the pancreas also have unique architecture, which helps them to accomplish their functional goal maintaining constant blood glucose. The multicellular system is composed mainly of three cell types: insulin-secreting *β* cells, glucagon-secreting *α* cells, and somatostatin-secreting *δ* cells. Insulin and glucagon are reciprocal hormones that decrease and increase blood glucose, respectively. Interestingly, different species have different islet architectures [2–5]. Mouse islets have a shell-core structure in which *β* cells are located in the core, while non-*β* cells are located in the periphery, surrounding the core. However, large human islets, which contain a lower fraction of *β* cells, have a mixing structure in which non-*β* cells are not only distributed throughout the islet periphery but also scattered within the islets. Recently, we have found that the spatial organization of islet cells follows a conserved rule in which homotypic cell-cell contacts have a slightly stronger attraction than heterotypic contacts [6].

Insulin, glucagon, and somatostatin secretions are pulsatile, like other endocrine hormones. The three pulses are not independent, but coordinated: the approximate out-of-phase coordination of insulin and glucagon pulses has been observed in the blood of normal humans but not in the blood of diabetic patients [7, 8]. Perifused islets have also shown out-of-phase coordination as well as the in-phase coordination of insulin and somatostatin at high glucose [9, 10]. Phase coordination implies that there is communication between islet cells. Indeed, this communication has been extensively studied in the form of paracrine/autocrine interactions via hormones and neurotransmitters [11].

**Fig 1:**
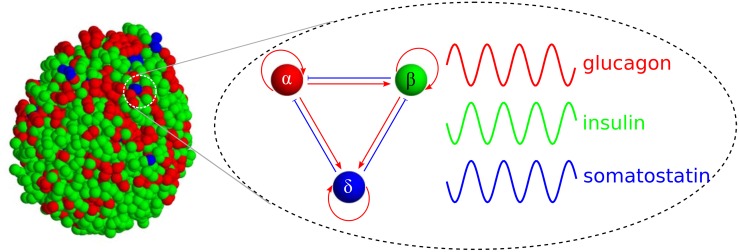
Cellular organization and interaction in pancreatic islets. Endocrine *α* (red), *β* (green), and *δ* cells (blue) generate pulses of glucagon, insulin, and somatostatin, respectively. They positively (red arrows) or negatively affect (blue bar-headed arrows) hormone pulses of neighboring cells.

Considering the mechanism of cellular communication, the spatial organization of islet cells should have functional implications. Clustered *β* cells secrete insulin more robustly than single *β* cells [12, 13]. Recent studies have demonstrated the effectiveness of *β*-cell clustering by systematically controlling the size of *β*-cell aggregates [14, 15]. However, to understand the organization of *α*, *β*, and *δ* cells beyond *β*-cell clustering, we await technical innovations that allow the identification of different cell types within islets and the recording of their activities at a high resolution. Nevertheless, we know (i) how single *α*, *β*, and *δ* cells produce hormone pulses [16], (ii) how the hormone pulses are affected by other hormone pulses [16], and (iii) how those cells are spatially distributed within islets [6]. We were motivated to integrate the model and the data (Fig 1), and we computationally inferred the functional implications of the islet architecture. In this computational study, we found that the organization of islet cells and their interactions are designed to produce synchronous hormone pulses under low- and high-glucose conditions and asynchronous hormone pulses under normal glucose conditions. We also observed that the controllable synchronization was deteriorated in diabetic islets.

## Islet model

We formulated an islet model on the basis of two observations:

1. Islet cells are intrinsic oscillators that produce pulses of endocrine hormones.
2. Islet cells interact with neighboring cells via paracrine/autocrine signaling.

First, insulin pulses have been widely observed in blood samples obtained from live animals [17] as well as perfused pancreata [18] and islets [9]. Pulsatility is an intrinsic property of *β* cells because isolated *β* cells can still generate oscillations of intracellular calcium concentration [19, 20], a trigger of insulin secretion. Isolated *α* cells [21] and *δ* cells [22] can also generate calcium oscillations. Second, islet cells secrete hormones and/or neurotransmitters, and the signaling molecules affect the hormone secretions of neighboring cells. The paracrine/autocrine interactions between *α*, *β*, and *δ* cells are summarized in Table 1.

**Table 1:** Interaction signs between islet cells.

Symbol	Interaction	Sign	Reference
*A*_*αα*_	*α* → *α*	+	[24–27]
*A*_*βα*_	*α* → *β*	+	[28–31]
*A*_*δα*_	*α* → *δ*	+	[32–34]
*A*_*αβ*_	*β* → *α*	−	[35–42]
*A*_*ββ*_	*β* → *β*	+	[43–46]
*A*_*δβ*_	*β* → *δ*	+	[47–49]
*A*_*αδ*_	*δ* → *α*	−	[35, 50–52]
*A*_*βδ*_	*δ* → *β*	−	[35, 50–53]
*A*_*δδ*_	*δ* → *δ*	⋅	⋅

To obtain a robust conclusion independent of the details of the model, we generated a minimal model that incorporates the two basic observations. The dynamics of coupled oscillators have been extensively studied using the prototypic model, the Kuramoto model [23]. Thus, we adopted an oscillator model:

$$\begin{array}{c} {\dot{\theta }}_{i}=\omega_{i}+\sum_{j\in \Lambda_{i}}K_{\sigma_{i}\sigma_{j}}\sin\left(\theta_{j}-\theta_{i}\right), \end{array}$$ (1)

where $\theta_{i}\in R$ and *σ*_*i*_ ∈ {*α*, *β*, *δ*} are the phase and type of the *i*th cell among *N* cells within an islet. The phase represents the degree of hormone secretion: given amplitude, *θ* = 0 and *θ* = *π* represent minimal and maximal secretion, respectively. Each cell produces oscillatory hormone secretion with an intrinsic frequency *ω*_*i*_. In this study, we focused on the oscillation with a period of *ω*^−1^ ∼ 10 minutes. For simplicity, we assumed that every cell had an identical frequency *ω*_*i*_ = *ω*; this assumption was relaxed later.

The second term in Eq (1) represents the interactions of the nearest neighboring *j*th cells. The neighborhood set Λ_*i*_ of the *i*th cell was predetermined from the data of the islet structures. The strength of the interaction from the *j*th cell to the *i*th cell is

$$\begin{array}{c} K_{\sigma_{i}\sigma_{j}}=A_{\sigma_{i}\sigma_{j}}r_{\sigma_{j}}{r_{\sigma_{i}}}^{-1} \end{array}$$ (2)

where *A*_*σ*_*i*_*σ*_*j*__ defines the sign of the interaction (Table 1) and *r*_*σ*_*i*__ and *r*_*σ*_*j*__ represent the relative activities of the receiver and affecter cells. Positive/negative interactions (*A*_*σ*_*i*_*σ*_*j*__ = ±1) lead the *i*th cell to have in-phase/out-of-phase oscillations with the *j*th cell.

The interactions are mediated by signaling molecules secreted from *α*, *β*, and *δ* cells. Thus, the interaction strengths should be dependent on the activities of cells that are governed by glucose level. One can consider those activities as average hormone secretions at different glucose concentrations [54, 55]. In general, *α* cells are active at low glucose, while *β* and *δ* cells are active at high glucose. Here we included the glucose conditions implicitly in the activities of islet cells, and simply defined low (*r*_*α*_ > *r*_*β*_), normal (*r*_*α*_ = *r*_*β*_), and high glucose conditions (*r*_*α*_ < *r*_*β*_). Thus *r*_*β*_/*r*_*α*_ is a proxy parameter for glucose conditions of which scale can be different from real glucose concentrations. The current *K*_*σ*_*i*_*σ*_*j*__ considers the activities of both affecter and receiver cells; the pair of a strong affecter and a weak receiver exhibits maximal coupling. We considered an alternative activity-dependent interaction, *K*_*σ*_*i*_*σ*_*j*__ = *A*_*σ*_*i*_*σ*_*j*__ *r*_*σ*_*j*__, which ignores the activity of receiver cells. Because these two cases did not show significant differences, we focused on the former definition in Eq (2). This setting helps to reduce the number of parameters from nine *K*_*σσ*′_ to three *r*_*σ*_. Next, we have a constraint, |*K*_*σ*_*i*_*σ*_*j*__| = |*K*_*σ*_*j*_*σ*_*i*__|^−1^, and this implies that every autocrine interaction has a unity of strength, |*K*_*αα*_| = |*K*_*ββ*_| = |*K*_*δδ*_| = 1.

## Results

### Islet cells generate the glucose-dependent coordination of insulin and glucagon pulses

We started by considering simple islets that had only *α* and *β* cells, two major cell populations (>90%). Considering the antagonistic roles of the two cell types for glucose homeostasis, they are likely to inhibit each other. Unexpectedly, however, they showed an asymmetric interaction rather than mutual inhibition: *β* cells indeed suppressed *α* cells (*K*_*αβ*_ < 0), but *α* cells stimulated *β* cells (*K*_*βα*_ > 0). We examined how the asymmetric interaction affected the coordination of hormone pulses within islets.

**Fig 2:**
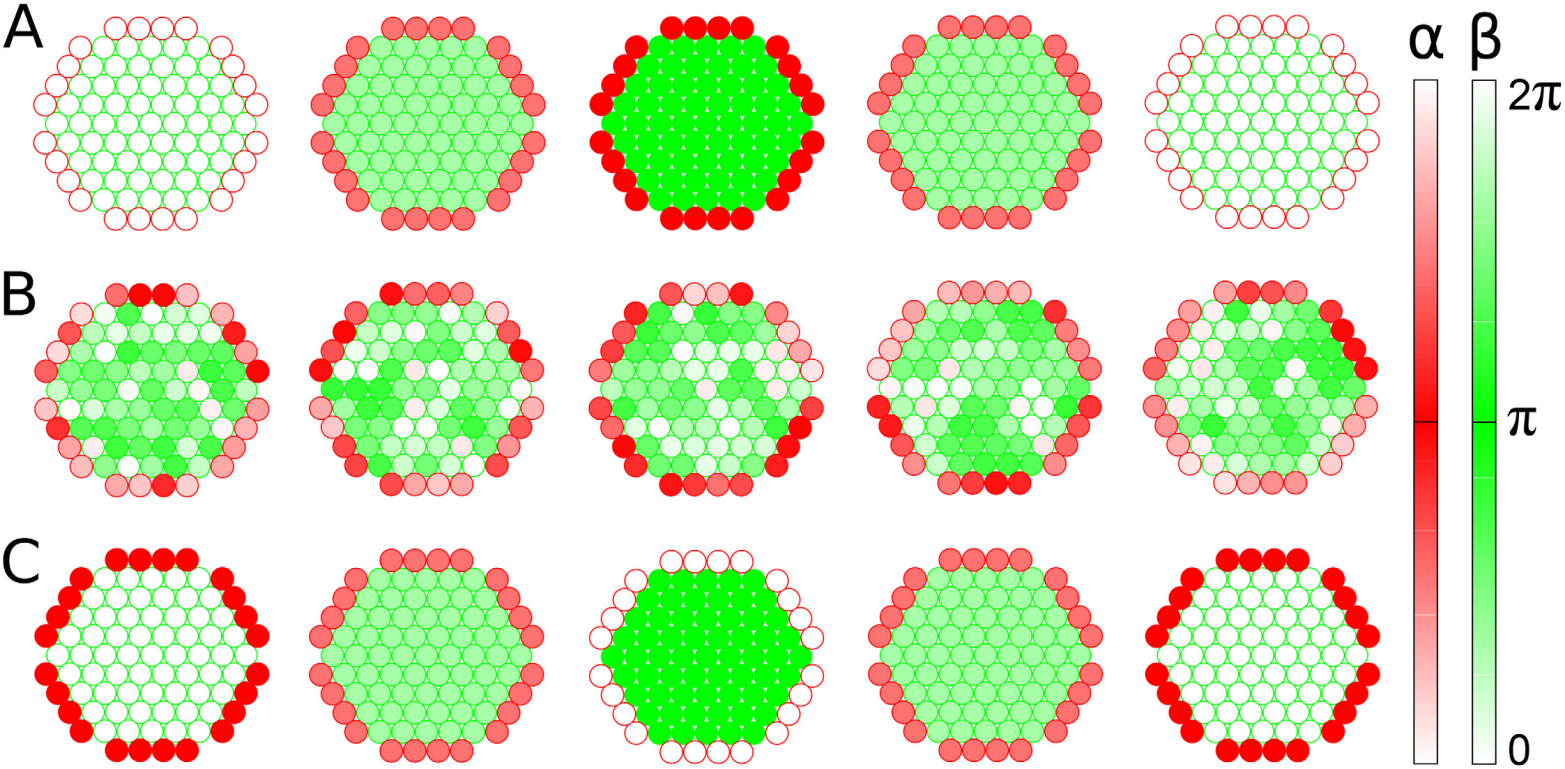
Snapshots of islet-cell activities. Sequential phase changes of *α* (red circle) and *β* cells (green circle) with time at different glucose conditions: (A) *r*_*β*_/*r*_*α*_ = 0.1 (low glucose); (B) *r*_*β*_/*r*_*α*_ = 1 (normal glucose); (C) *r*_*β*_/*r*_*α*_ = 10 (high glucose). Each cell spontaneously alternates its phase between 0 (light color) and *π* (dark color), and its neighboring cells perturb the oscillation. Note that cross sections of three-dimensional structures are displayed for clarity.

First, we simulated the dynamics of *α* and *β* cells in Eq (1) with a prototypic organization of core *β* cells and peripheral *α* cells. The multicellular system had three equilibrium states, depending on the glucose conditions:

1. In-phase synchronous state. When *α* cells were active (*r*_*α*_ > *r*_*β*_) at low glucose, they secreted neurotransmitters, which sensitized *β* cells to secrete insulin [31]. Here the positive interaction (*K*_*βα*_ = *r*_*α*_/*r*_*β*_) from *α* to *β* cells dominated the negative interaction (*K*_*αβ*_ = −*r*_*β*_/*r*_*α*_) from *β* to *α* cells. In addition, the positive autocrine interactions (*K*_*αα*_ = *K*_*ββ*_ = 1) helped homotypic cells synchronize with each other. Given these conditions, synchronous *α* cells were coordinated in phase with synchronous *β* cells (Fig 2A and S1 Video). This state represented the in-phase coordination of glucagon and insulin pulses.
2. Asynchronous state. When *α* and *β* cells were equally active (*r*_*α*_ = *r*_*β*_) at normal glucose, the asymmetric interaction had the same strength (|*K*_*βα*_| = |*K*_*αβ*_| = 1). The *α* cells were “confused” about whether to be active because neighboring *α* cells activated them but neighboring *β* cells equally suppressed them. This incongruous condition ultimately resulted in both *α* and *β* cells becoming asynchronous, although local homotypic clusters temporally showed synchronous behaviors (Fig 2B and S2 Video). Local synchronization has been observed in a recent experimental study [56].
3. Out-of-phase synchronous state. When *β* cells were active (*r*_*α*_ < *r*_*β*_) at high glucose, they secreted insulin and neurotransmitters, which suppressed *α* cells from secreting glucagon. Unlike the low-glucose condition, the negative interaction (*K*_*αβ*_ = −*r*_*β*_/*r*_*α*_) from *β* to *α* cells dominated the positive interaction (*K*_*βα*_ = *r*_*α*_/*r*_*β*_) from *α* to *β* cells. Thus, the synchronous *α* cells were coordinated out of phase with the synchronous *β* cells (Fig 2C and S3 Video). This state represented the out-of-phase coordination of glucagon and insulin pulses, which has been repeatedly reported [7–10].

The three states were not sharply divided but smoothly altered with glucose conditions (*r*_*β*_/*r*_*α*_). Thus, we introduced order parameters *R*_*α*_ and *R*_*β*_ that measured the degree of synchronization between *α* cells and *β* cells, respectively (See Materials and Methods): *R*_*α*_ = 1 and 0 represent perfect synchronization and desynchronization between *α* cells, respectively. The same is true for *R*_*β*_. In addition, we measured the phase difference ΔΘ between average *α*-cell phase Θ_*α*_ and *β*-cell phase Θ_*β*_: ΔΘ = 0 and *π* represent perfect in-phase and out-of-phase states, respectively.

**Fig 3:**
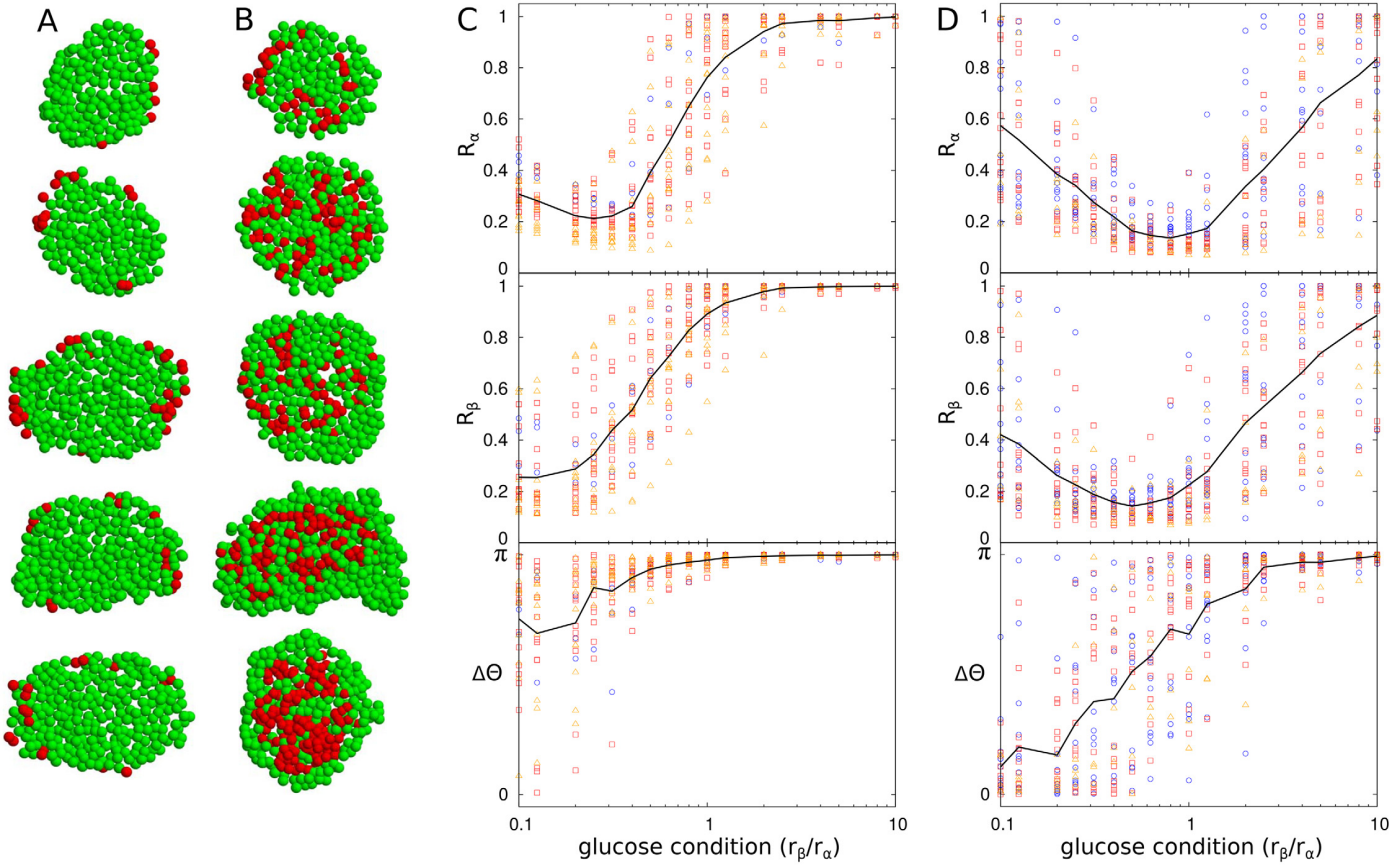
Glucose-dependent synchronization of islet cells in mouse and human islets. Cross sections of (A) mouse and (B) human islets with *α* (red) and *β* cells (green). Synchronization and phase coordination of islet cells in (C) mouse (n = 29) and (D) human islets (n = 28). Synchronization indices *R*_*α*_ and *R*_*β*_ represent the degrees of synchronization between *α* cells and between *β* cells, respectively, and phase index ΔΘ indicates the difference of average phases of *α* and *β* cells. Islets are categorized into three groups according to size *N*: small islets (*N* < 1000 cells, blue circle); medium islets (1000 < *N* < 2000, red square); and large islets (*N* > 2000, orange triangle). Black lines represent average values of corresponding indices of every islet.

Using these order parameters, we examined the dynamics of *α* and *β* cells that interacted given the spatial distributions in mouse and human islets (Fig 3A and 3B). We observed the above three states frequently in the two species. However, the out-of-phase synchronous state was more pronounced in mouse islets that had more *β* cells (Fig 3C), while the in-phase synchronous state was more pronounced in human islets that had more *α* cells (Fig 3D). Different sizes of islets showed similar dynamics in both mouse and human islets. The size independence is of particular interest in human islets that have different cellular compositions according to their size. Interestingly, *α* cells and *β* cells were partially synchronous (*R*_*α*_ < 1 and *R*_*β*_ < 1), except under very high-glucose conditions. In particular, the in-phase synchronous state at low glucose was largely suppressed in mouse islets. Asynchronous oscillation of *α* cells at low glucose has been experimentally observed [57].

Pancreatic islets contain other cell types. Although *δ* cells compose a minor portion of the population (<10%), they can affect the cellular dynamics because they interact with *α* and *β* cells (Table 1), as *δ* cells suppress both *α* and *β* cells, but are activated by both *α* and *β* cells. To probe the role of *δ* cells, we compared the cellular dynamics in the presence and absence of *δ* cells within islets (S1 Fig). The complex interactions between *α*, *β*, and *δ* cells disrupted the synchronizations of *α* and *β* cells. In general, the existence of *δ* cells decreased the degree of synchronization, but the minor population did not dramatically modify the above results that ignored *δ* cells. Thus, for simplicity, we do not consider *δ* cells hereafter.

### The organizations and interactions of islet cells are designed for smooth transitions between synchronous and asynchronous hormone pulses

**Fig 4:**
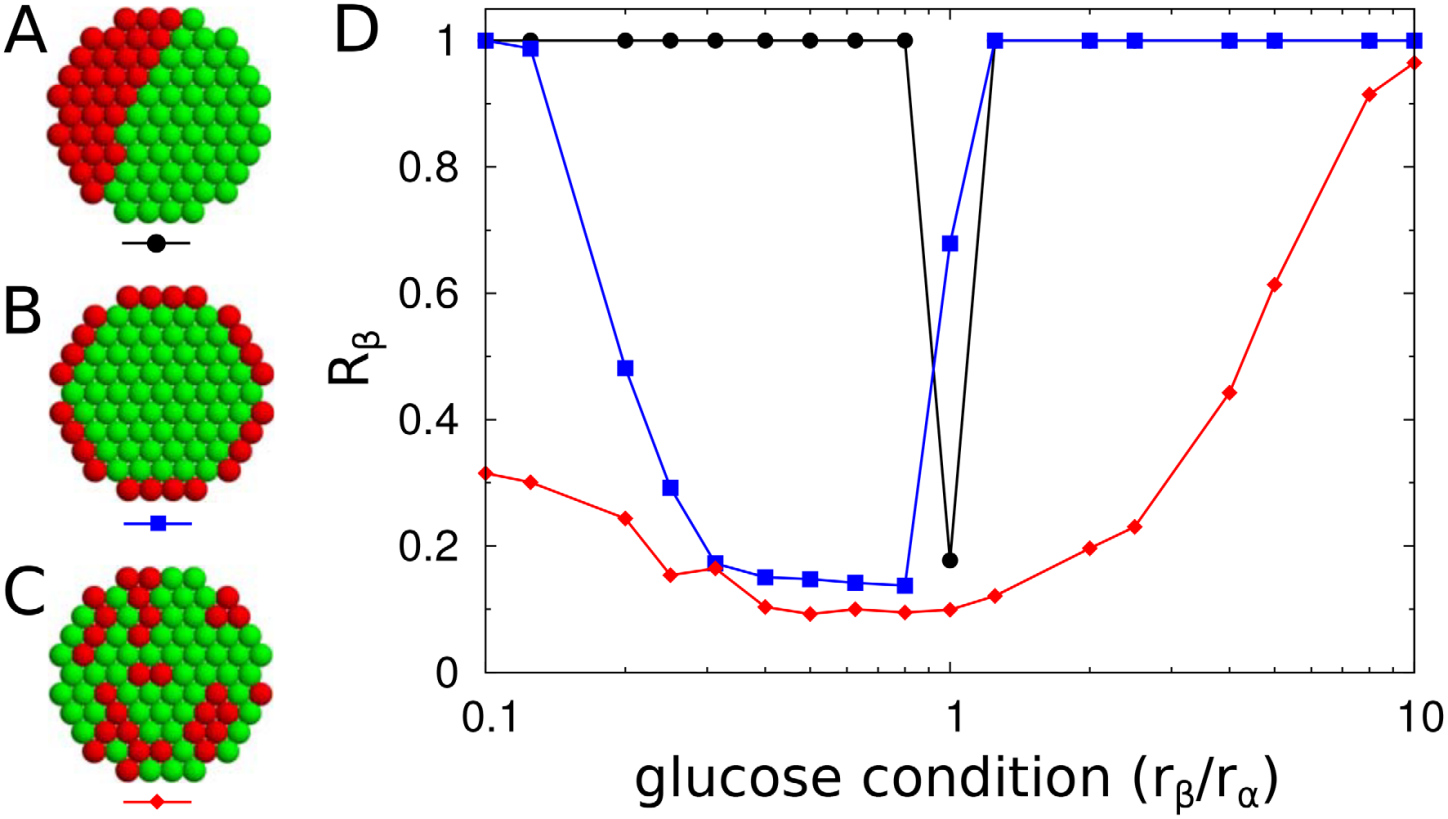
Islet structure and synchronization. (A) Complete sorting (black circle), (B) shell-core sorting (blue square), and (C) mixing (red diamond) structures of *α* (red) and *β* cells (green). The total number of cells and the fraction of *β* cells are fixed as *N* = 725 and *p*_*β*_ = 0.6, respectively for all three structures. Note that cross-sections of three-dimensional structures are displayed for clarity. (D) Synchronization index *R*_*β*_ of *β* cells for different glucose conditions.

To systematically investigate the design principles of natural islets, we considered artificial islets that had different organizations of islet cells or different interactions between them. As a backbone for the three-dimensional arrangement of islet cells, we adopted hexagonal-close-packed lattices [6, 58] and controlled the spatial distributions and compositions of *α* and *β* cells, (*p*_*α*_ and *p*_*β*_, respectively). We generated different islet structures by tuning the relative adhesions between cell types (See Materials and Methods). Three distinct structures were (i) the complete sorting structure, in which homogeneous cell clusters were divided into left and right hemispheres; (ii) the shell-core sorting structure, in which *β* cells were clustered in the core and *α* cells were in the periphery; and (iii) the mixing structure, in which *α* and *β* cells were intermingled with each other. For a fixed cellular composition (*p*_*α*_ = 0.4, *p*_*β*_ = 0.6), the three structures showed different patterns of synchronization (Fig 4). Unlike the shell-core and mixing structures, the complete sorting structure always generated perfect synchronization between cells except under a very narrow glucose condition (*r*_*β*_/*r*_*α*_ = 1). The lack of partial synchronization resulted in abrupt transitions between the synchronous and asynchronous states.

**Fig 5:**
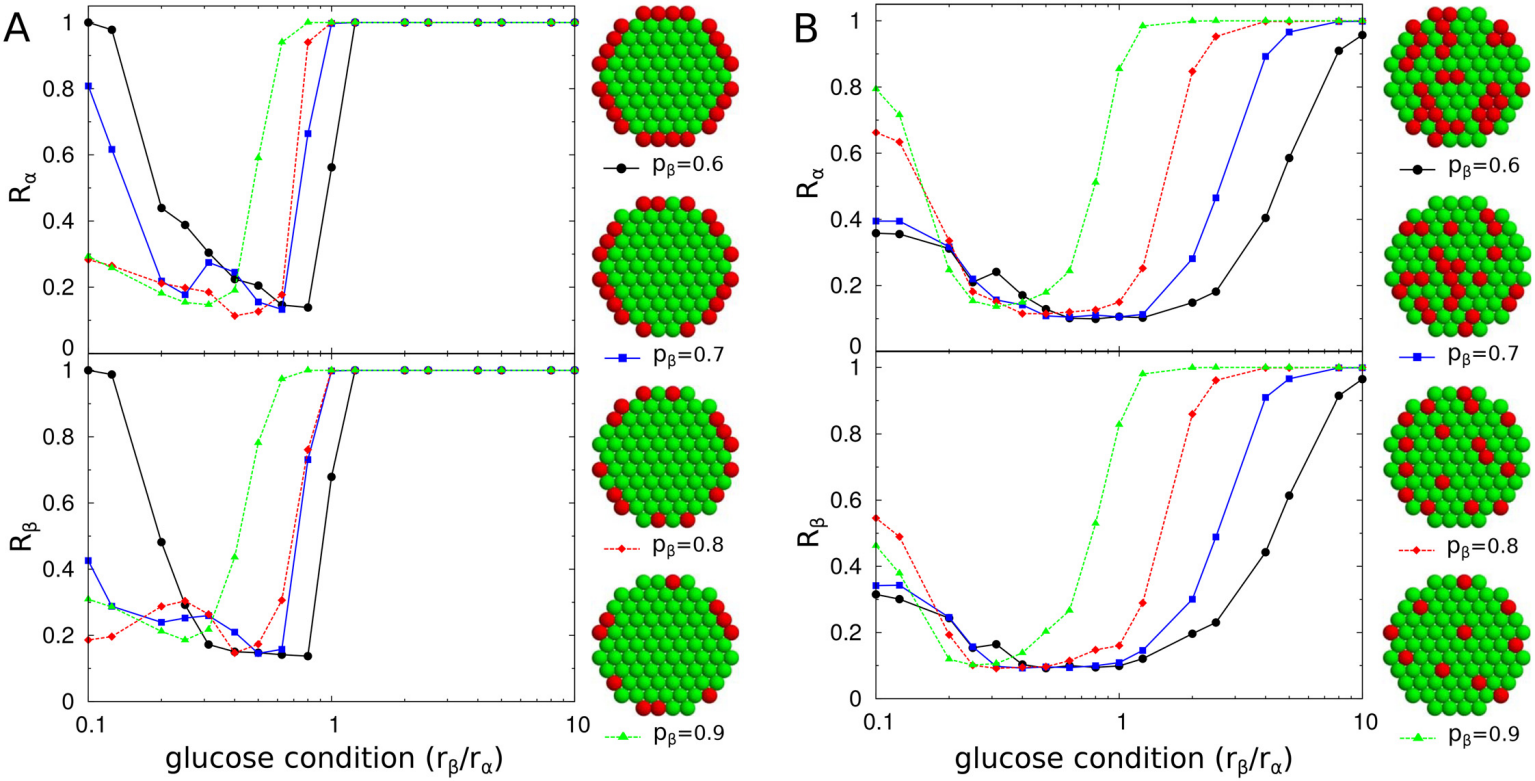
Cellular composition and synchronization. Synchronization indices *R*_*α*_ of *α* cells (red) and *R*_*β*_ of *β* cells (green) for various cellular compositions in (A) shell-core sorting and (B) mixing structures. The fractions of *β* cells are *p*_*β*_ = 0.6 (black circle), 0.7 (blue square), 0.8 (red diamond), and 0.9 (green triangle) among *N* = 725 cells. Note that cross-sections of three-dimensional structures are displayed for clarity.

Next, we controlled cellular compositions given a total cell number (*N* = 725). Mouse islets have a shell-core structure with a high fraction of *β* cells (*p*_*β*_ ≈ 0.9). If the *β*-cell fraction was decreased in mouse islets, then the multi-cellular dynamics showed an enhancement of the in-phase synchronous state and sharper slopes of *R*_*α*_ and *R*_*β*_ with respect to the glucose conditions (Fig 5A). However, human islets have the mixing structure with a smaller fraction of *β* cells (*p*_*β*_ ≈ 0.6). If the *β*-cell fraction was increased in human islets like the fraction in mouse islets, then the modified human islets had the enhanced in-phase synchronization and sharper slopes of *R*_*α*_ and *R*_*β*_ (Fig 5B). Therefore, the large *β*-cell fraction in mouse islets and small *β*-cell fraction in human islets are effective in suppressing the in-phase synchronization between insulin and glucagon pulses, and in preventing sharp transitions between the synchronous and asynchronous hormone pulses as glucose conditions change. These conclusions were the same for different sizes of islets (S2 Fig).

The opposite dependence of *β*-cell fractions for mouse and human islets was related to the number and size inhomogeneity of *α*-cell clusters. The number of *α* cells was not sufficient to form a large cluster in the shell-core structure, so several clusters of *α* cells that varied in size existed. However, as the number of *α* cells increased in the mixing structure, clusters of *α* cells of various sizes started to appear. The separate clusters of *α* cells contributed to the diminishing of the synchronization of *α* cells.

**Fig 6:**
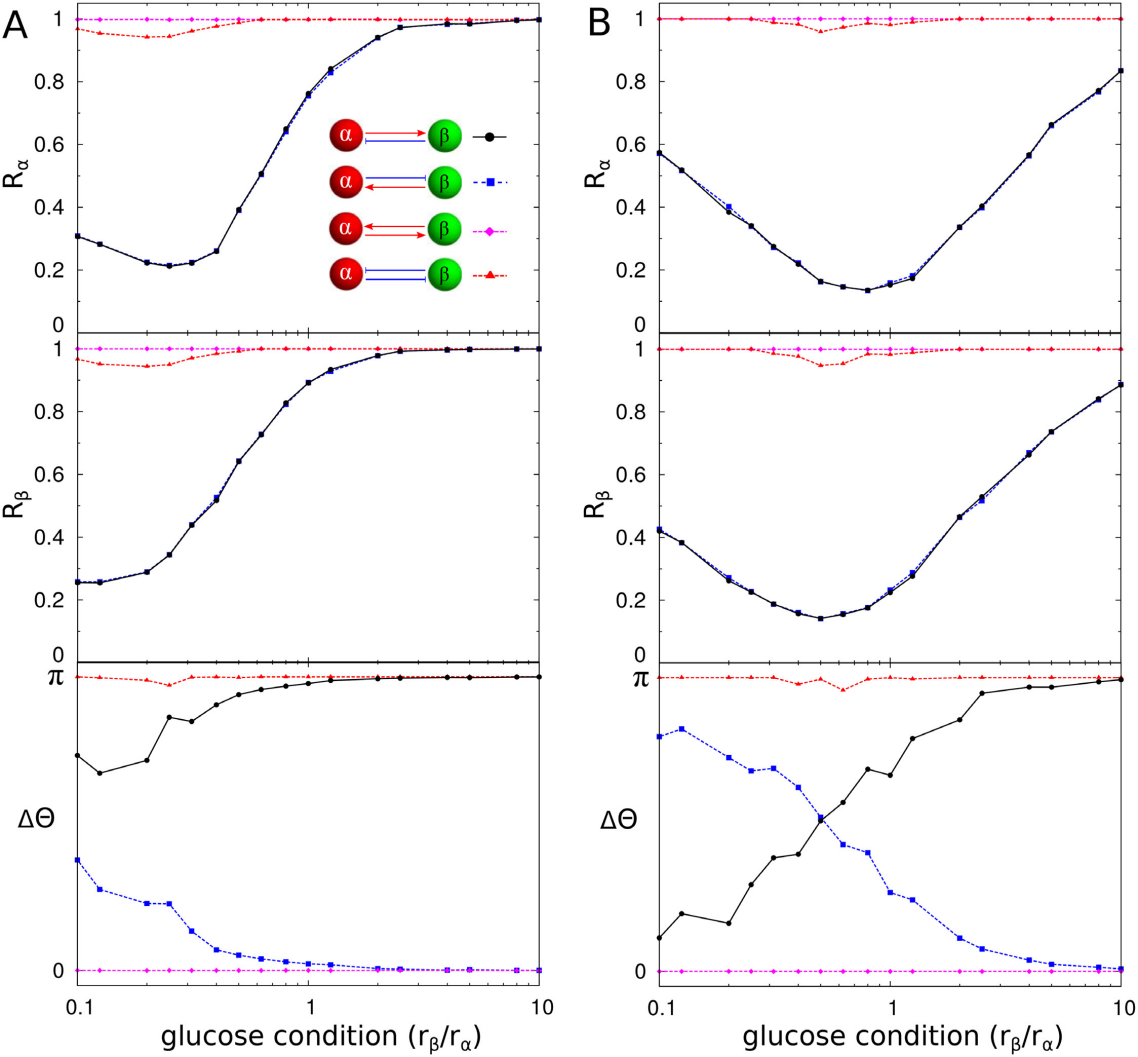
Cellular interaction and synchronization. Synchronization indices *R*_*α*_ of *α* cells (red) and *R*_*β*_ of *β* cells (green) and the average phase difference ΔΘ between *α* and *β* cells are measured for four scenarios of the mutual interaction between *α* and *β* cells in (A) mouse and (B) human islets: (i) *α* cells activate *β* cells, while *β* cells suppress *α* cells (black circle); (ii) opposite interaction to (i) (blue square); (iii) mutual activation (magenta diamond); and (iv) mutual inhibition (red triangle). Note that (i) black line represents the result from the true interaction in natural islets (See Fig 3).

Next, we modified the interactions between *α* and *β* cells by considering every possibility in regard to their mutual interaction. In natural islets, *α* cells sensitize *β* cells, while *β* cells suppress *α* cells. Unlike the asymmetric interaction, when the two cells symmetrically activated or inhibited each other, they always generated synchronous hormone pulses that were independent of the glucose conditions (Fig 6). However, the mutual activation model always showed in-phase coordination of insulin and glucagon pulses, while the mutual inhibition model always showed out-of-phase coordination of the pulses. When the asymmetric interaction was reversed, the controllability of the synchronization was intact, but the phase coordination of insulin and glucagon pulses was reversed for the glucose conditions. If *α* cells inhibited *β* cells and *β* cells activated *α* cells, then they could generate the in-phase coordination of insulin and glucagon pulses under high-glucose conditions.

### Diabetic islets fail to produce coordinated pulses of insulin and glucagon

**Fig 7:**
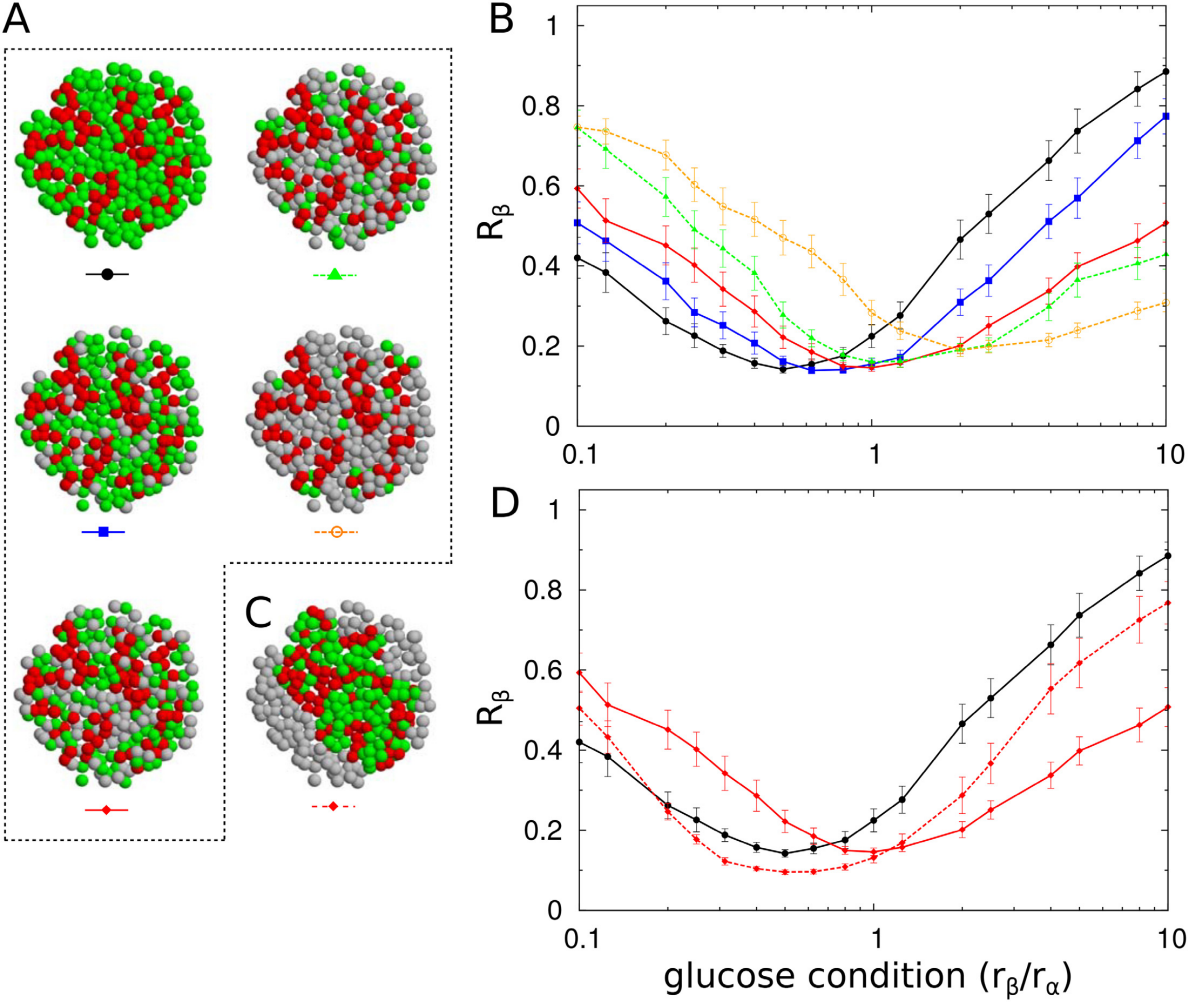
Synchronization of islet-cells under *β*-cell loss. (A) To simulate human diabetic islets, *β* cells were selectively removed randomly from human islets (n = 28): no (black circle), 30% (blue square), 50% (red diamond), 70% (green triangle), and 90% loss of *β*-cell mass (yellow empty circle). Note that cross-sections of three-dimensional structures are displayed for clarity. (B) Synchronization index *R*_*β*_ of *β* cells for the loss of *β* cells. (C) The remaining cells after the removed *β* cells (50%) were re-aggregated. (D) Synchronization index *R*_*β*_ of *β* cells with (red diamond, solid line) and without (red diamond, dashed line) the consideration of re-aggregation. The error bars represent standard errors.

We simulated diabetic islets by removing *β* cells (Fig 7A). The random removal of *β* cells attenuated the synchronization of islet cells under high-glucose conditions (Fig 7B). In particular, a drastic change was found when approximately 50% of the *β* cells were removed. A loss of out-of-phase coordination of insulin and glucagon pulses has been observed in diabetic patients [7]. Furthermore, *α* cells became relatively abundant due to the selective loss of *β* cells. This change strengthened the positive interactions from *α* cells, which then enhanced the synchronization of islet cells under low-glucose conditions.

To more realistically simulate the diabetic islets, the re-aggregation of remaining cells should be considered (Fig 7C), because the sites of removed *β* cells could not physically remain as an empty space. One interesting question is whether the space-filling is just a passive process or whether it can be an active process used to escape the deteriorated condition. When we considered re-aggregation (See Materials and Methods), the enhanced synchronization at low glucose and suppressed synchronization at high glucose were partially recovered to original patterns for normal islets (Fig 7D). This finding suggested that the re-organization of islet cells under diabetic conditions could actively contribute to recovery.

## Discussion

We computationally studied the design principles of pancreatic islets by integrating their structure and function in a model. Our model incorporated the pulsatility of hormone secretions, paracrine/autocrine interactions, and spatial organization of pancreatic *α*, *β*, and *δ* cells. We found that the multicellular system functions not only to produce synchronous hormone pulses at low/high glucose but also to produce asynchronous hormone pulses under normal glucose conditions. The controllable synchronization effectively enhanced and suppressed hormone actions, depending on the glucose conditions. Thus, we proposed that the defective controllability of hormone pulses could contribute to metabolic diseases, such as diabetes.

We predicted the glucose-dependent coordination of hormone pulses. Regarding the phase coordination of insulin and glucagon pulses, previous studies have reported out-of-phase [7, 9], in-phase [59, 60], and no [18, 61] relationships between the two pulses. Controversies could originate from variations in animal species, islet preparation, and/or experiment conductors. Our finding, however, implies that the different phase coordinations could be partially explained by the different glucose conditions in the studies.

We adopted a minimal model with essential ingredients to reduce unnecessary complexities and obtain robust conclusions. Indeed, we confirmed that some possible modifications did not change our conclusion about controllable synchronization (S3 Fig). First, we used an alternative form of paracrine/autocrine interactions (*K*_*σ*_*i*_*σ*_*j*__ = *A*_*σ*_*i*_*σ*_*j*__ *r*_*σ*_*j*__) that ignored the activity of receiver cells. Second, we relaxed the assumption that every cell had the same intrinsic frequencies by introducing some variations of *w*_*i*_ ∈ [0.8, 1.2]. Third, we applied a stronger interaction (*K*_*ββ*_ = 2) between *β* cells as the simplest way to consider their gap-junctional coupling in addition to their autocrine interaction. Finally, we ignored the autocrine interaction of *δ* cells (*K*_*δδ*_ = 0), because this interaction has not been observed yet; its contribution is expected to be negligible because contact between *δ* cells is very rare.

Nevertheless, the minimal model was limited to incorporating all of the observed complexities of the system. In the phase-oscillator model, we simplified the shape of hormone pulses as a sine wave, although their ridge and valley durations could depend on the glucose conditions. Next, we reduced the number of parameters that described the strengths of the paracrine/autocrine interactions by assuming that they were governed by the activities of the cells. The reduced degrees of freedom may have constrained the multicellular system from generating richer dynamics. Thus we cannot rule out the possibility that *δ* cells play a crucial role in the multicellular system. For example, the out-of-phase coordination of glucagon and insulin pulses at high glucose may be predominantly led by the inhibitory interaction from somatostatin rather than insulin [62, 63]. Third, the paracrine interactions may depend on external glucose stimuli as well as the activities of cells. The stimulatory interaction from *α* cells to *β* cells via glucagon functions in the presence of glucose stimuli [64]. Forth, the sign of the interactions may not be fixed. Indeed, the sign of the autocrine interaction of *β* cells remains controversial [65]. Finally, although we focused on the short-range interactions of islet cells with no time delay, they may have long-range interactions via blood vessels and nerves densely innervated in islets. However, current experimental data are not comprehensive enough to probe the relevance of these complexities to the controllability of a multicellular system.

We thus conclude that pancreatic islets have a special design that allows to control (enhance/suppress) hormone actions depending on glucose conditions. Our finding of islet structure and function can suggest new perspectives for the diagnosis and therapy of diabetes. For example, the controllability of synchronization between hormone pulses can be a novel measure for the functionality of pancreatic islets. Furthermore, our theoretical model can provide an optimal structure of artificial islets made by stem cells.

In living systems, desynchronization is as important as synchronization [66, 67]. Pancreatic islets showed one possible design for controllable synchronization. The design principle can be applied to other multicellular systems such as neural networks that have both excitatory and inhibitory connections.

## Materials and methods

### Quantification of synchronization

The degree of synchronization between cells in the same population is characterized by a generalized order parameter [68]:

$$\begin{array}{c} R_{\sigma }e^{i2\Theta_{\sigma }}=\frac{\sum_{j=1}^{N}\delta_{\sigma ,\sigma_{j}}e^{i2\theta_{j}}}{\sum_{j=1}^{N}\delta_{\sigma ,\sigma_{j}}}, \end{array}$$ (3)

where the amplitude *R*_*σ*_ (0 ≤ *R*_*σ*_ ≤ 1) measures the phase coherence of *σ* ∈ {*α*, *β*, *δ*} cells, and the phase Θ_*σ*_ represents the average phase of *σ* cells. Here the Kronecker delta function, *δ*_*σ*, *σ*_*j*__, represents that the *j*th cell contributes with *δ*_*σ*, *σ*_*j*__ = 1 only when its type is *σ*, otherwise *δ*_*σ*, *σ*_*j*__ = 0. Notably, we used a multiplication factor of 2 for the order parameters because the multicellular dynamics showed that cells in the same population were sometimes divided into two groups with a phase difference *π* (See S1 Text). The usual order parameter without the multiplication factor could not distinguish this ordered condition from a completely disordered one.

### Structure of mouse and human islets

We used the structural information of mouse and human islets from our previous study [6]. Briefly, isolated mouse (*n* = 29) and human (*n* = 28) islets were stained with glucagon and insulin antibodies, and the three-dimensional coordinations of individual cells within single islets were identified using a confocal microscope. Then, the neighborhood of each cell was identified by calculating the cell-to-cell distances. In six samples of human islets, *δ* cells stained with the somatostatin antibody were identified as well as *α* and *β* cells. The cell coordinate data are available in Supporting Information (S1, S2 and S3 Datasets).

### Simulation of islet organization and reorganization

To examine design principles of the natural organization of islet cells, we generated their artificial organization. We used hexagonal close-packed (HCP) lattices as a backbone structure for the artificial islet organization [6]. The spatial organization of islet cells was determined by minimizing the total cell-to-cell adhesion energy,

$$\begin{array}{c} E=-\frac{1}{2}\sum_{i=1}^{N}\sum_{j\in \Lambda_{i}}J_{\sigma_{i}\sigma_{j}}, \end{array}$$ (4)

where *J*_*σ*_*i*_*σ*_*j*__ represents the adhesion energy for the contact of *i*th cell and *j*th cell with their corresponding cell types, *σ*_*i*_ and *σ*_*j*_ ∈ {*α*, *β*, *δ*}; and Λ_*i*_ denotes the set of nearest neighboring cells of the *i*th cell. The adhesion model could generate various structures, depending on the parameter set *J*_*σσ*^′^_: complete sorting structure (*J*_*αα*_ = 1, *J*_*αβ*_ = 0.5, *J*_*ββ*_ = 1), shell-core sorting structure (*J*_*αα*_ = 1, *J*_*αβ*_ = 1.5, *J*_*ββ*_ = 3), and mixing structure (*J*_*αα*_ = 1, *J*_*αβ*_ = 0.98, *J*_*ββ*_ = 1), given a total *N* = 725 cells [6]. Briefly, we (i) randomly distributed the numbers of *α* and *β* cells on HCP lattices; (ii) randomly chose two cells to swap, and calculated the total adhesion energies, *E* and *E*^′^, before and after exchanging their positions; (iii) accepted the exchange with the probability, min[1,*e*^(*E*−*E*^′^)/*T*^], following the Metropolis algorithm [69], where the parameter *T* = 0.2 controls the fluctuation of cellular organizations; and (iv) repeated these procedures in several million Monte-Carlo steps per cell to obtain an equilibrium structure.

We applied the adhesion model to simulate the re-aggregation of the remaining cells in diabetic islets. We considered empty sites of removed *β* cells as $\bar{\beta }$ cells. Then, the adhesion parameters *J*_*αα*_ = *J*_*ββ*_ = 1, *J*_*αβ*_ = 0.98, and $J_{\alpha \bar{\beta }}=J_{\beta \bar{\beta }}=J_{\bar{\beta }\bar{\beta }}=0$ could simulate the re-aggregation in diabetic islets because cells prefer contacting with cells to contacting with empty sites (See S4 Video).

### Numerical integration

To integrate Eq (1), we used the Euler method [70] with a sufficiently-small time step Δ*t* = 0.01. The intrinsic frequency could be set to *ω* = 0 for convenience because the multicellular dynamics is invariant under the transformation, *θ*_*i*_(*t*)−*ωt* → *θ*_*i*_(*t*). The initial phases $\theta_{i}\left(0\right)\in R$ were randomly chosen. The quantities of interest, such as the complex order parameters, were measured after a sufficiently long transient was discarded.

## Supporting Information

S1 Video Dynamics of islet-cell activities. Low-glucose condition (*r*_*β*_/*r*_*α*_ = 0.1). Activities (or phases) of *α* (red circle) and *β* cells (green circle) change with time. Each cell spontaneously alternates its phase between 0 (light color) and *π* (dark color), and its neighboring cells perturbs the oscillation given cellular interactions. Note that cross-sections of three-dimensional structures are displayed for clarity.
(AVI)

S2 Video Dynamics of islet-cell activities. Normal glucose condition (*r*_*β*_/*r*_*α*_ = 1).
(AVI)

S3 Video Dynamics of islet-cell activities. High-glucose condition (*r*_*β*_/*r*_*α*_ = 10).
(AVI)

S4 Video Re-aggregation simulation of islet cells. Under 50% removal of *β* cells (green), the remaining *α* (red) and *β* cells re-aggregate given preferential contacts between cells (See Materials and Methods). Note that this simulation was conducted on a two-dimensional lattice for clarity, but the re-aggregation simulations for Fig 7C and 7D were conducted on three-dimensional lattices.
(AVI)

S1 Text Model of four coupled oscillators. We introduce a simple case where cells in the same population can be divided into two groups with a phase difference *π* under multicellular dynamics in Eq 1.
(PDF)

S1 Fig Role of *δ* cells for islet-cell synchronization. An islet structure in (A) the presence and (B) absence of *δ* cells. Note that cross sections of three-dimensional structures are displayed for clarity. (C) Synchronization index *R*_*β*_ of *β* cells is plotted in the presence (black filled circle) and absence (blue empty circle) of *δ* cells. The error bars represent standard errors of the mean (n = 6). For the simulation, *r*_*α*_ = *r*_*δ*_ = 1 and *r*_*β*_ ∈ [0.1, 10] were used.
(EPS)

S2 Fig Islet size and synchronization. Synchronization index *R*_*β*_ of *β* cells for three islet sizes with shell-core (left column) and mixing structures (right): (A) and (B) *N* = 725 (top row), (C) and (D) 1357 (middle), and (E) and (F) 2493 (bottom) hexagonal-close-packed lattices. Different cellular compositions are considered as Fig 5. The fractions of *β* cells are *p*_*β*_ = 0.6 (black circle), 0.7 (blue square), 0.8 (red diamond), and 0.9 (green triangle). The data resulted from averages of five ensembles using different initial conditions for solving Eq 1.
(EPS)

S3 Fig Model robustness. Synchronization index *R*_*β*_ of *β* cells was examined under modifications (blue empty circle, dotted line) of the original model (black filled circle, solid line). (A) Strength of cellular interactions, |*K*_*σσ*′_| = *r*′_*σ*_ vs. |*K*_*σσ*′_| = *r*_*σ*′_/*r*_*σ*_ (Fig 3D). (B) Intrinsic frequency, *ω*_*i*_ = [0.8, 1.2] vs. *ω*_*i*_ = 1 (Fig 3D). (C) Stronger interaction between *β* cells, *K*_*ββ*_ = 2 vs. *K*_*ββ*_ = 1 (Fig 3D). (D) No interaction between *δ* cells, *K*_*δδ*_ = 0 vs. *K*_*δδ*_ = 1 (S1 Fig).
(EPS)

S1 Dataset Islet structure data. Three-dimensional coordinates of *α* and *β* cells within mouse islets (n = 29). Columns represent types, x, y, and z coordinates (*μ*m) of cells (rows) within an islet. The numbers of the first column are 11 (*α* cell) and 12 (*β* cell).
(ZIP)

S2 Dataset Islet structure data. Three-dimensional coordinates of *α* and *β* cells within human islets (n = 28). Columns represent types, x, y, and z coordinates (*μ*m) of cells (rows) within an islet. The numbers of the first column are 11 (*α* cell) and 12 (*β* cell).
(ZIP)

S3 Dataset Islet structure data. Three-dimensional coordinates of *α*, *β*, and *δ* cells within human islets (n = 6). Columns represent types, x, y, and z coordinates (*μ*m) of cells (rows) within an islet. The numbers of the first column are 11 (*α* cell), 12 (*β* cell), and 13 (*δ* cell).
(ZIP)
