# Supplementary material for: Design Principles of Pancreatic Islets: Glucose-Dependent Coordination of Hormone Pulses
Source: PLoS One. 2016 Apr 1;11(4):e0152446. doi: 10.1371/journal.pone.0152446 (PMC4818077; doi:10.1371/journal.pone.0152446)
Supplement: S1 Text — We introduce a simple case where cells in the same population can be divided into two groups with a phase difference π under multicellular dynamics in Eq 1. (PDF) [file pone.0152446.s005.pdf]

Text S1

Design Principles of Pancreatic Islets: Glucose-dependent Coordination of Hormone Pulses

Danh-Tai Hoang[1,2], Manami Hara[3], Junghyo Jo[1,4,*]

[1]Asia Pacific Center for Theoretical Physics, Pohang, Gyeongbuk 36763, Korea
[2]Department of Science, Quang Binh University, Dong Hoi, Quang Binh 510000, Vietnam
[3]Department of Medicine, The University of Chicago, Chicago, IL 60637, United States of America
[4]Department of Physics, Pohang University of Science and Technology, Pohang, Gyeongbuk 36763, Korea

* jojunghyo@apctp.org

MODEL OF FOUR COUPLED OSCILLATORS

We considered a simple setup to demonstrate the importance of network structure on multicellular dynamics. Suppose we put two types of cells on a one-dimensional array with four sites. Here, individual cells generate intrinsic oscillations and affect the oscillations of their nearest neighboring cells. The phase dynamics of the interacting cells on the four sites is described by

$$\dot{\theta}_1 = \omega + K_{\sigma_1\sigma_2}\sin(\theta_2 - \theta_1), \tag{A.1}$$
$$\dot{\theta}_2 = \omega + K_{\sigma_2\sigma_1}\sin(\theta_1 - \theta_2) + K_{\sigma_2\sigma_3}\sin(\theta_3 - \theta_2), \tag{A.2}$$
$$\dot{\theta}_3 = \omega + K_{\sigma_3\sigma_2}\sin(\theta_2 - \theta_3) + K_{\sigma_3\sigma_4}\sin(\theta_4 - \theta_3), \tag{A.3}$$
$$\dot{\theta}_4 = \omega + K_{\sigma_4\sigma_3}\sin(\theta_3 - \theta_4), \tag{A.4}$$

where w is their intrinsic frequency, σ_i is the cell type on the ith site, and $K_{\sigma_i\sigma_j}$ represents the interaction from the jth cell to the ith cell which depends on their cell types. Because we are interested in the relative phases between cells, we define them: $x \equiv \theta_1 - \theta_2$, $y \equiv \theta_2 - \theta_3$, and $z \equiv \theta_3 - \theta_4$. Then, we obtain the equations of motion for the relative phases from the four phase equations:

$$\dot{x} = -(K_{\sigma_1\sigma_2} + K_{\sigma_2\sigma_1})\sin x + K_{\sigma_2\sigma_3}\sin y, \tag{A.5}$$
$$\dot{y} = K_{\sigma_2\sigma_1}\sin x - (K_{\sigma_2\sigma_3} + K_{\sigma_3\sigma_2})\sin y + K_{\sigma_3\sigma_4}\sin z, \tag{A.6}$$
$$\dot{z} = K_{\sigma_3\sigma_2}\sin y - (K_{\sigma_3\sigma_4} + K_{\sigma_4\sigma_3})\sin z. \tag{A.7}$$

Here, $x_*, y_*, z_* \in \{0, \pi\}$ are stationary solutions of Eqs. (A.5)-(A.7). To examine their stabilities, we can linearize $\sin(x_* + \epsilon_x) \approx S_x\epsilon_x$ around the solutions x_*, where $S_x = 1$ for $x_* = 0$, and $S_x = -1$ for $x_* = \pi$. The same is true for y_* and z_*. Defining a vector $\boldsymbol{\epsilon} = (\epsilon_x, \epsilon_y, \epsilon_z)$, we obtained a linear equation $\dot{\boldsymbol{\epsilon}} = \boldsymbol{K}\boldsymbol{\epsilon}$ with

$$\boldsymbol{K} \equiv \begin{bmatrix} -(K_{\sigma_1\sigma_2} + K_{\sigma_2\sigma_1})S_x & K_{\sigma_2\sigma_3}S_y & 0 \\ K_{\sigma_2\sigma_1}S_x & -(K_{\sigma_2\sigma_3} + K_{\sigma_3\sigma_2})S_y & K_{\sigma_3\sigma_4}S_z \\ 0 & K_{\sigma_3\sigma_2}S_y & -(K_{\sigma_3\sigma_4} + K_{\sigma_4\sigma_3})S_z \end{bmatrix}. \tag{A.8}$$

Next, we confirmed the stabilities of the eight stationary solutions, $x_*, y_*, z_* \in \{0, \pi\}$, by examining the sign of the eigenvalues of the matrix $\boldsymbol{K}$. We have two α cells and two β cells with different arrangements (Fig. A.1). The strength of cellular interactions is governed by the activities r_{σ_j} and r_{σ_i} of affecter and receiver cells: $K_{\sigma_i\sigma_j} \equiv A_{\sigma_i\sigma_j} r_{\sigma_j} r_{\sigma_i}^{-1}$. The signs of the interactions are encoded in $A_{\alpha\alpha} = A_{\beta\beta} = 1$, $A_{\alpha\beta} = -1$, and $A_{\beta\alpha} = 1$. Given the activities r_{σ_j} and r_{σ_i}, different cell arrangements have different stable stationary states (Fig. A.1).

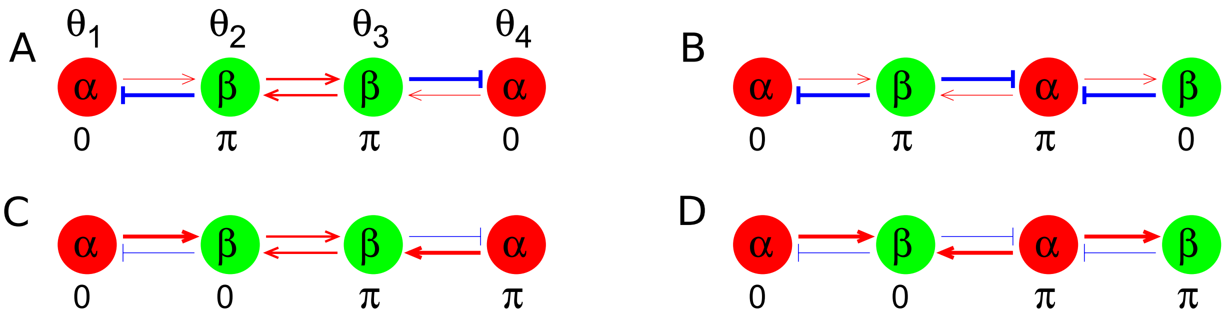

FIG. A.1. **Synchronization of four coupled islet cells.** (A) Shell-core and (B) mixing arrangements of two α (red) and two β cells (green). Numbers below cells represent stable stationary phases at high glucose ($r_\beta/r_\alpha = 10$). Note that the phase of the first site is set to be $\theta_1 = 0$ for simplicity. (C) Shell-core and (D) mixing arrangements at low glucose ($r_\beta/r_\alpha = 0.1$). Arrows (red) represent positive interactions, while bar-headed arrows (blue) represent negative interactions. The thickness of arrows depicts the relative strength of cellular interactions given the glucose conditions.
